# Experiences of Siblings of Children With Neurodevelopmental Disorders: Comparing Qualitative Analysis and Machine Learning to Study Narratives

**DOI:** 10.3389/fpsyt.2022.719598

**Published:** 2022-04-28

**Authors:** Jort. A. J. Bastiaansen, Elien E. Veldhuizen, Kees De Schepper, Floortje E. Scheepers

**Affiliations:** Department of Psychiatry, University Medical Center Utrecht, Utrecht, Netherlands

**Keywords:** child, adolescent, sibling, narratives, machine learning, topic modeling, natural language processing, neurodevelopmental disorders

## Abstract

**Introduction:**

Relatively few studies have focused on the wellbeing, experiences and needs of the siblings of children with a psychiatric diagnosis. However, the studies that have been conducted suggest that the impact of such circumstances on these siblings is significant. Studying narratives of diagnosed children or relatives has proven to be a successful approach to gain insights that could help improve care. Only a few attempts have been made to study narratives in psychiatry utilizing a machine learning approach.

**Method:**

In this current study, 13 narratives of the experiences of siblings of children with a neurodevelopmental disorders were collected through largely unstructured interviews. The interviews were analyzed using the traditional qualitative, hermeneutic phenomenology method as well as latent Dirichlet allocation (LDA), an unsupervised machine learning method clustering words from documents into topics. One aim of this study was to evaluate the experiences of the siblings in order to find leads to improve care and support for these siblings. Furthermore, the outcomes of both analyses were compared to evaluate the role of machine learning in analyzing narratives.

**Results:**

Qualitative analysis of the interviews led to the formulation of nine main themes: confrontation with conflicts, coping strategies siblings, need for rest and time for myself, need for support and attention from personal circle, wish for normality, influence on personal choices and possibilities for development, doing things together, recommendations and advices, ambivalence and loyalty. Using unsupervised machine learning (LDA) 24 topics were formed that mostly overlapped with the qualitative themes found. Both the qualitative analysis and the LDA analysis detected themes that were unique to the respective analysis.

**Conclusion:**

The present study found that studying narratives of siblings of children with a neurodevelopmental disorder contributes to a better understanding of the subjects' experiences. Siblings cope with ambivalent feelings toward their brother or sister and this emotional conflict often leads to adapted behavior. Several coping strategies are developed to deal with the behavior of their brother or sister like seeking support or ignoring. Devoted support, time and attention from close relatives, especially parents, is needed. The LDA analysis didn't appear useful to distract meaning and context from the narratives, but it was proposed that machine learning could be a valuable and quick addition to the traditional qualitative methods by finding overlooked topics and giving a rudimental overview of topics in narratives.

## Introduction

This study aims to get insights into the experiences of siblings of children with neurodevelopmental disorders, using both qualitative and machine learning methods of analysis. Additionally, this study intents to compare these forms of analysis. The worldwide prevalence of psychiatric disorders in children and adolescents is estimated to be 13.4% (1), with attention deficit hyperactivity disorder (ADHD) being the third most prevalent disorder (3.4%). The prevalence of autism spectrum disorder (ASD) is estimated 1–2% amongst children worldwide (2). In the Netherlands, where this study was conducted, approximately 57% of the households have more than one child or adolescent (3). Therefore, when a clinician diagnoses a child with a psychiatric disorder, there is a high chance of the diagnosed child having one or more siblings in his or her family. It is common to involve family members in the treatment of children and adolescents–for example by providing psychoeducation–to promote the health and development of the child with a diagnosis. Despite this, there is still much to learn about the impact of neurodevelopmental disorders on the siblings in the family, including on the siblings' wellbeing and needs. Having more knowledge about the experiences and needs of siblings could contribute to the improvement of their own personal wellbeing, as well as the wellbeing of the diagnosed child or adolescent.

So far studies on this topic that have been conducted, mostly focused on siblings of children with an autism spectrum disorder. They show that being a sibling of a child or adolescent with ASD has negative aspects, but can also have a positive influence. Positive aspects that are mentioned are having a brother or sister with a good nature (fun, loving, humor), growing up more mature and increased social competence. Negative aspects include the disruptive behavior itself, being embarrassed by such behavior and internalizing feelings (4, 5). Some studies evaluate the functioning of siblings of children with autism spectrum disorder. Vieira and Fernandes (6) studied quality of life of siblings using the World Health Organization Quality of Life questionnaire and did not find a lower quality of life. One study focusing on the quality of life by Moyson and Roeyers (7) did not quantify and compare the quality, but explored the extensive impact on the life of siblings of patients with ASD. Examples of the impact were, among others, having to deal with aggressive behavior and the lack of private time. In addition, siblings seem to be vulnerable to developing psychiatric symptoms themselves. Ma et al. (8) found an increased overall risk for psychiatric symptoms in siblings of patients, especially patients with attention deficit hyperactivity disorder. On the other hand, Regalla et al. (9) found that siblings of patients with ADHD had greater resilience when assessed with the Resilience Scale. In a small pilot study Callio and Gustafsson (10) found that siblings of patients with eating disorders deal with concerns and lack of information about their siblings' disorder, although it led to better awareness of a healthy diet. No studies specifically on the experiences of children with siblings that are diagnosed with are psychiatric disorders like depression, anxiety or schizophrenia were found. Sin et al. (11) interviewed 31 siblings of patients with a first psychosis, of their participants 7 were under 16 years old. The young participants report dealing with tensions in the family and coping by keeping a low profile. The younger siblings mention the need for respite care aimed at keeping their own lives and studies as normal as possible amidst the disruption often caused by having a brother or sister with early psychosis in the household. Some studies focused on the narratives of adults looking back on growing up with a sibling with a psychiatric disorder. Lukens et al. (12) held focus groups with 19 adults that grew up with siblings diagnosed with schizophrenia, bipolar disorder and major depression. The respondents portrayed complex feelings of fear, anger, guilt, loss, and loneliness that they associated with the presence of an emerging and chronic mental illness in a brother or sister.

There is a risk of overlooking the needs of the siblings because of the behavior and needs of the diagnosed brother or sister. Dyregrov and Dyregrov (13) wrote about “the forgotten bereaved”; children and adolescents who lose a sibling due to suicide. It was found that only 40% of the siblings received community service after the death, while two-thirds of the parents said it would have been preferable if the siblings had received help. Fox, Dean, and Whittlesea (14) conclude in their systematic review of the impact of having an eating disorder on the family that the needs of siblings are often overlooked by clinicians and researchers. One would assume that providing professional support to siblings would be helpful, but so far evidence is lacking that support groups for s iblings are effective (15).

The aim of this study is to gain insights into the experiences of siblings of children with neurodevelopmental disorders such as ASD, ADHD and intellectual disability using a narrative approach. Experiences are defined as the way that something happens and how it makes someone feel (16). Analyzing personal, daily experiences and stories of recovery contribute to the improvement of healthcare, giving us more insight into the social, political and rights aspects of illness and recovery (17). It was found that narratives can promote recovery and provide support for patients with limited access to peers (18). Hermeneutic phenomenology is a method of analyzing narratives by focusing on the meaning of experiences (19) derived from the philosophy of Ricoeur (20). It has been used several times to study narratives of parents with children with a psychiatric disorder (21) and narratives of siblings of children with a psychiatric disorder (7, 22).

Rather than quantitative information, narratives provide us with detailed and individual information about the personal and lived experience. However, they are often difficult to study because of their unstructured nature (23). Qualitative research is the traditional approach to analyze unstructured texts or narratives, often leading to new insights and forming new hypotheses from interviews and texts. Qualitative research is labor intensive, especially when analyzing large sets of texts. With increasing computing power and new big data strategies, the field of natural language processing and text mining is emerging. These techniques could be a helpful instrument to study narratives. The strategies can be roughly divided into supervised machine learning, where an algorithm is trained with known inputs and outputs to make predictions, and unsupervised machine learning where the algorithm defines clusters in data without knowing the output variable. A review by Abbe et al. (24) revealed that different text mining strategies have been studied in mental health. They found that text mining has been used to identify psychopathology, gain knowledge about patient perspective, improve electronical medical records and to explore the expanding medical literature. The majority of studies Abbe et al. included used supervised machine learning–for example to identify symptoms of a depression or extract Mini-Mental State Examination scores from an unstructured text. Only a few studies looked at unsupervised machine learning techniques to evaluate the meaning of psychiatric narratives or texts.

For this current study the value of using unsupervised machine learning to detect topics in interviews was compared to traditional manual qualitative analysis. This was done using topic modeling via LDA, first described by Blei et al. (25). Some studies have explored topic modeling in psychiatry before. Carron-Arthur et al. (26) used LDA to find topics in mental health internet support groups and compared topics between frequent users and less frequent users; finding structural and substantive similarities between the internet help group and a professional support group. Aufegger et al. (27) studied the feasibility of using LDA to improve patient safety management by analyzing transcribed focus groups they extracted four themes, amongst which hospital culture and the different responsibilities between team members. Using topic modeling to analyze transcribed psychiatric consultations in patients with psychotic disorders did not turn out to be a tool to predict psychotic symptoms. However, it was possible to predict evaluations of the therapeutic relationship (28). The same study also compared hand coded topics and topics formed by machine learning, which revealed an overlap in topics. LDA topics seemed to pick up additional factors to the contact (for example, negative and positive aspects of communication vs. general relationship). A trained LDA model can also be used to analyze new content and predict patient characteristics. This was studied by Valenti and Bock (29) who trained an LDA topic model to predict the emotional state of Parkinson's patients after being interviewed and calculated a correct prediction in 86% of the interviews. Barroilhet et al. (30) used LDA topic modeling to characterize personality traits of a psychiatric inpatient cohort based on electronic health notes.

The first aim of the current study is to evaluate the experiences of siblings of children with a neurodevelopmental disorder. What is the impact of having a sibling with a neurodevelopmental disorder and how can healthcare be improved from their personal experiences? It was hypothesized that using narratives would give insight into the needs of the siblings that might be overlooked. In this way new leads to improve care and support for these siblings can be pointed out. A second aim was to test the feasibility of using unsupervised machine learning to analyze narratives and compare its results to traditional analyses. Our hypothesis was that machine learning could provide a more neutral perspective of the narratives, revealing information that might have been overlooked by an individual researcher who might be influenced by personal interpretations.

## Materials and Methods

### Participants

The current study is part of the Stories Database Psychiatry initiative (*Verhalenbank Psychiatrie*) of the Utrecht University Medical Center that collects narratives of patients, relatives and professionals. This initiative aims to analyze personal narratives to improve patient care, but also to provide a platform where patients and relatives can share their stories with each other and find support. It has been modeled after the Dementia Stories Database (*Dementie Verhalenbank*) (31). For this study we collected personal narratives of siblings of children with a psychiatric disorder in order to get insights into the impact of having a diagnosed sibling and to see if it provides us with leads to improve care for the whole family. Participants were recruited between March and May 2018 in two ways. First, via parents of under-age patients who received treatment at the University Medical Center Utrecht (UMC Utrecht). Invitation letters were handed out to parents of children who were clinically treated at the acute psychiatry ward and the developmental disorder ward, as well as parents of children who attended the developmental disorder day treatment. Secondly, participants were recruited via parents who attended a lecture on the effect of developmental disorders on the direct environment in 2017 organized by UMC Utrecht. Thirdly, a call for participants was placed on the website of the Brain Division of UMC Utrecht and the website of parent association Balans. Inclusion criteria were age (8 to 16 years old) and having an under-age sibling diagnosed with a psychiatric disorder. Participants were excluded when they were diagnosed with a psychiatric disorder themselves. The age, gender, family situation of the participant and the sibling were collected, as well as and diagnosis of the sibling. This project was approved by the Medical Ethical commission of the UMC Utrecht and written informed consent was obtained from participants and both their parents.

### Demographics

Thirteen participants responded to the invitation letter. All thirteen participants met the inclusion criteria and none were excluded. The demographics of the participants and the siblings are outlined in Tables 1, 2. Six participants were recruited from the visitors of the lecture, seven participants were siblings of patients that were treated at the developmental disorder day treatment (five participants) or the developmental disorder clinic (two participants) of UMC Utrecht. No participants were recruited via the patients of the acute clinic and no participants were recruited via the call on the websites. All participants had a sibling diagnosed with a developmental disorder (ASD or ADHD). Some siblings had multiple diagnoses (e.g., one sibling also had depressive symptoms, one sibling also had an intellectual disability). Unfortunately, no participants with siblings with other diagnoses (like eating disorders, psychosis or anxiety disorder) were included. The average age of the participants was 10.5 years with a distribution from 8 to 15 years. Six participants were female and seven participants were male. The average age of the diagnosed siblings was 11.3 years, and 80% were male. Four participants had divorced parents and lived with their mother, the other participants lived with both parents. Three participants had more than one sibling with a psychiatric disorder. Two participants (brothers) came from the same family. The participants lived in the provinces of Gelderland, Noord-Holland and Utrecht (The Netherlands).

**Table 1:** Demographics of participating siblings.

**Variable**	**Sibling (*n =* 13)**
Age, average (distribution)	10.5 (8–15)
Sex, n (%)	
- Male	7 (54%)
- Female	6 (46%)
Position in the family, n (%)	
- First	5 (38%)
- Second	6 (46%)
- Third or more	2 (16%)
Position in relation to diagnosed sibling	
- Older	7 (50%) ^*^
- Younger	7 (50%) ^*^

* *One participant had two diagnosed siblings, older and younger*.

**Table 2:** Demographics of diagnosed brothers and sisters.

**Variable**	**(Diagnosed) children (n = 15)**
Average age of diagnosed sibling (distribution)	11.3 (7–17)
Sex, n (%)
- Male	12 (80%)
- Female	3 (20%)
Number of psychiatric diagnosis of siblings (some siblings had multiple diagnosis)	
- Attention deficit hyperactivity disorder	3 (20%)
- Autism spectrum disorder	9 (60%)
- Unspecified disruptive, impulse-control, and conduct disorder	1 (7%)
- Major depressive disorder	1 (7%)
- Intellectual disability	1 (7%)

### Study Design

The study has a qualitative research design, as we were most interested in understanding the experience of being a sibling from their own perspective. The interviews were conducted in Dutch. The interviews were individual, in-depth, unstructured and phenomenology-based, as described below. Due to the quality of the interviews being influenced by the degree of trust felt by the siblings, the following measures were taken to attain this trust. Siblings were given the opportunity to choose the place where the interview was conducted, a place where they felt most at ease to tell their story. A flexible topic list was used to give the siblings the opportunity to suggest their own topics. This also enabled the siblings not to discuss topics they found difficult or disliked talking about. Every interview started with relevant questions from a standardized set of generic opening questions regarding themselves (e.g., “What makes you happy?”, “What are your wishes?”, “Where do you go to school?”) to make the children feel comfortable before talking about their relationship with their siblings. The full list with questions and topics can be found in the Supplementary Material. The interviews lasted a maximum of 60 min, in which enough time was left for the researcher to interact with the child and gain their trust. This extra time proved to be important for the siblings to become familiar with the researcher.

### Analysis

The interviews were analyzed in two ways: first by hand, using the hermeneutic phenomenology method and secondly by machine learning using LDA. The LDA modeling was conducted by a different assessor than the qualitative analysis.

#### Hermeneutic Phenomenology Method

Hermeneutic phenomenology is a commonly used method to interpret the meaning of experiences from interviewees. It aims not only at finding phenomena (experiences), but also to evaluate and interpret the meaning of these experiences (19). This method has been used before to study narratives of parents with children with a psychiatric disorder (21). It has also been used to interpret experiences of siblings of children with psychiatric diagnoses, specifically of those with autism and intellectual disabilities (7, 22).

The phenomenological hermeneutical analysis consisted of three phases, in which we searched for recurring patterns (19). In the first phase, a basic understanding of the text was obtained from a global initial reading; ‘naïve reading’. The purpose was to get a holistic understanding of the text and the essential meaning of the stories told. What is being told? What does the sibling want to express? In the second phase, a structured analysis was done. The text was being read as objectively as possible, while adding categories to the text (e.g., metaphors, filler words, protagonists and time perspectives). Following this, the text was divided into meaning units. A meaning unit can be part of a sentence, a sentence, several sentences, a paragraph, i.e., a piece of any length that conveys just one meaning (19). The meaning units were used to extract overlapping meanings, which were collected as subthemes (e.g., feeling sad or angry, talking to parents, ambivalent feelings). The subthemes among the different interviews were used to create main themes (e.g., coping strategies, need for proximate support and attention, confrontation with conflicts). All the meaning units, subthemes and main themes were continuously validated against the background of the naïve reading to remain close to the essential message of the text and the actual lived experience. This makes the analysis a circular process. After a first assessment the text was read by a second assessor. All the analyses and codes were discussed with other members of the study group for a critical reflection. In the final phase, the meaning units, themes and sub themes are being summarized. The results were described as much as possible in the words and language of the participants and were formed taking into account the earlier phases (naïve reading and structured reading). After following these steps, the summarized results were used to answer the research questions.

#### Latent Dirichlet Allocation

**Figure 1:**
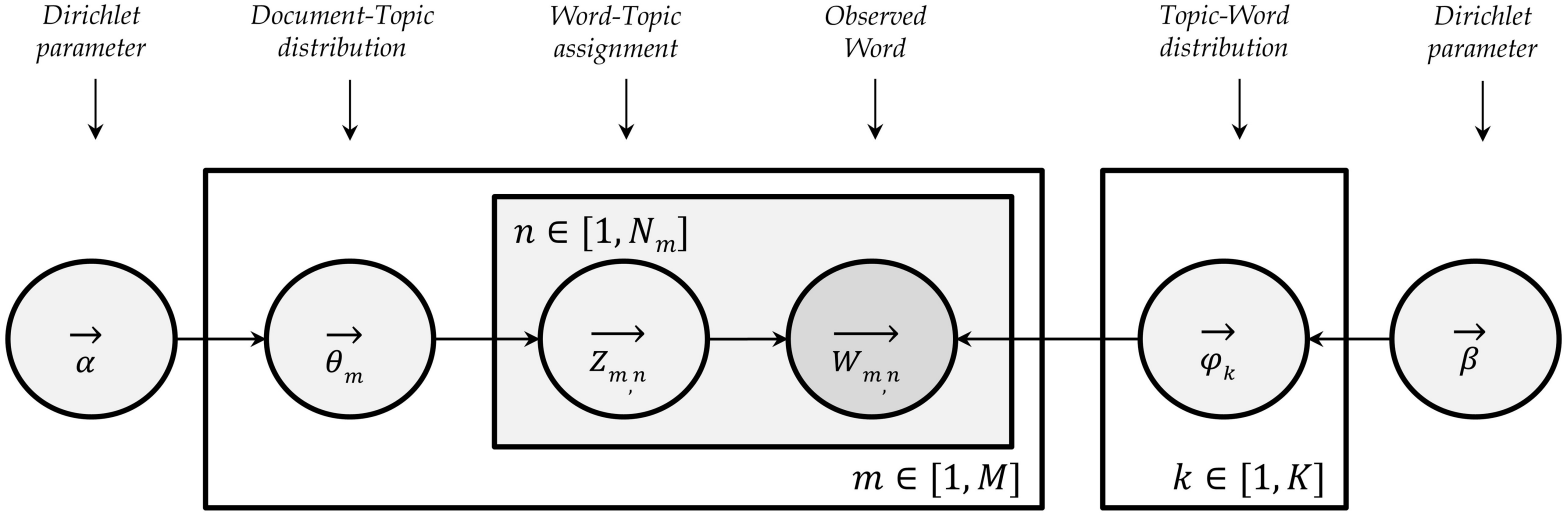
Graphical model of latent Dirichlet allocation. Shaped after a graphic by Lee et al. (33).

The computer assisted analysis of the interviews was conducted using LDA (25, 32). Figure 1 displays a graphical model of LDA. It calculates probabilities for words in a chosen amount of topics. In the current study the corpus is the whole set of interviews and the documents are the separate answers provided. The R package TextmineR was used to perform the analysis. Documentation about this package can be found online (34).

To perform this analysis the interviews were preprocessed, removing common Dutch filler words and personal names, and keeping only open class words (i.e., nouns, adjectives, verbs). Different lemmas from the same lexeme (e.g., go and going) were joined into one lemma. For the removal of filler words the R package *stopwords* was used (35); for the filtering and joining of lemmas the R package *udpipe* (36) was used. The 20 most frequently occurring words were inspected manually and words without a significant meaning or wrongly assigned as open class were removed to prevent the formation of meaningless topics. Words that appeared less than twice or appeared in more than half of the documents were also removed to prevent the model from including terms that are either too specific or not specific enough. Furthermore, both unigrams (single words) and bigrams (combination of two words) were considered, because bigrams can have a different meaning than the combination of separate terms and thus add value to the topics.

The LDA model needs a topic number as input (*k*). To compare the topics with the topics from the qualitative analysis, we aimed at 15 to 25 topics. A coherence score was calculated per total number of topics to evaluate in which model the topics had the highest probability to be related. For the definite topic model, the model with the highest coherence score was selected. In addition, the outcomes of the model with an extended topic range (15 to 35 topics) were screened to ensure this did not provide more intelligible topics. Another relevant variable in the model is *alpha*, which is a Dirichlet parameter for the distribution of topics in documents. Alpha was set to 0.05, lower than the default (0.1), since the documents used (the answers in the interview) were relatively short and were likely to contain just one or a few different topics.

LDA calculates clusters of words that have a probability of forming a topic when used together, but does not give the topic meaningful names. Therefore two child and youth psychiatrists were appointed to separately interpret the top 20 words per topic and assign labels to the topics. Following this, a consensus group was held to see which label fitted best. The quality of each topic was also addressed, scoring the topics as “easily intelligible” (+), “difficult to interpret” (+−) or “not intelligible” (−).

Moreover, the LDA model calculates *gamma* values, which calculate the probable distribution of topics per document. The mean distribution of the topics in the complete set of interviews were calculated, as well as the distribution per interview. The latter gave insight into which topics occurred most frequently in the interview and which topics occurred most frequently taking the average prevalence in account.

#### Comparison

The outcome of both analyses were manually compared to gauge whether the machine-clustered topics matched the manually coded themes.

## Results

### Hermeneutic Phenomenology Analysis

In the first structured analysis, narrative categories were determined (i.e., protagonists, time perspective, common stop words, metaphors). Protagonists in the interviews were the interviewees and their siblings with a disorder. Additionally, parents and other siblings were mentioned. The time perspective was mostly present tense, although sometimes the interviewees used past tense, mostly to describe their development relative to their sibling. Other themes discussed in past tense involved hospitalizations of the diagnosed sibling and the time period around the diagnosis of the psychiatric disorder. The future tense was mostly used to describe hopes and wishes for the future. Most participants used first-person pronouns, yet some participants switched to second-person pronouns when discussing difficult situations related to the sibling or when they expressed their own wishes and desires. The participants often used filler words (for example “or so”, “etcetera”) in the context of discussing disruptive behavior of the sibling, behavior of the parents toward the sibling or when expressing their own thoughts and feelings toward (the behavior of) the diagnosed sibling. Some younger participants gave evasive answers or did not reply to some questions. This seemed to be mainly the case with subjects related to their (negative) feelings, behavior or experiences related to their sibling.

In the second structured analysis all transcripts were coded with different themes that reflected parts of the text on the basis of recurring described experiences and observations. Examples included “disruptive behavior of the sibling”, “processing difficult feelings”, “mutual activities” and “negative emotions”. The codes formed meaning units, which are parts of the text that contain a specific important meaning. These were used to initially form subthemes and, in the third analysis, main themes. Examples of such themes in the third analysis included were “confrontation with conflicts” and “coping strategies of diagnosed siblings”. An overview of the nine main themes and subthemes of the final analysis can be found in Table 3. The table also contains a column with related LDA topics, which will be discussed in more detail in the next paragraph.

**Table 3:** Main and subthemes found with manual qualitative analysis, alongside corresponding LDA topics.

	**Main themes**	**Subthemes**	**Related LDA topic**
1	Confrontation with conflicts	- Psychical aggression - Verbal aggression - Busy behavior - Angry behavior - Unsolvable fights, unbeatable behavior - Seclusion, inaccessibility	- Fight - Siblings - Anger at home - Emotion
2	Coping strategies siblings	- Accepting behavior sibling an adjusting (own) behavior - Taking sibling in account and adjusting (own) behavior - Leave sibling alone - Ignoring disruptive behavior - Avoiding actions - Impulsive reactions - Seeking support from others - Positive attitude	- Going well - Siblings - Leaving alone - Giving attention
3	Need for rest and time for myself	- Not feeling like adjusting - Time for myself / private time with parents	- (Bed)room - Giving attention
4	Need for support and attention from personal circle	- Talking to parents - Talking to other significant persons - Having a hard time not to be able to go to parents - No need for professional, additional help	- Social network - Parents - Giving attention
5	Wish for normality	- Fantasy about live without sibling, with different sibling	*No related LDA topics*
6	Influence on personal choices and possibilities for development	- Influence on everyday personal choices - Influence on choices for the future - Influence on personal development	- Siblings
7	Doing things together	- Pay attention to desires, needs of and possibilities for sibling - Being dependent on behavior sibling - Appreciating sharing of positive experiences	- At home and out - Nice things - (Enjoying) sports - Vacation - Taking into account
8	Recommendations and advices	- Clarity in communication to sibling - Positivity and tranquility - Ignoring disruptive behavior / not taking it personally - Approach fights in the form of a game - Seeking help from parent	- Parents - Leaving alone - Going well - Talk to understand
9	Ambivalence and loyalty	- Ambivalent feelings - Loyalty to parents and sibling	- Opinion (II) - Giving attention - Emotion

The qualitative analysis extracted several main themes and subthemes from the interviews. In all narratives, conflicts and fights *(theme 1)* with siblings were mentioned, which often lead to verbal or physical aggression. As a result of the diagnosed brother or sister often lacking insight into his or her own behavior, it is difficult for their siblings to address the behavior. Some siblings mention that these fights can make them sad, frustrated or angry. Furthermore, some siblings describe that conflicts can seem unsolvable, which leads to feelings of powerlessness and frustration.

It is remarkable that all siblings demonstrated a certain level of reflection on how they react to the disruptive behavior of the diagnosed brother or sister. They adjust themselves behaviorally, cognitively and emotionally to the circumstances to meet the needs of the diagnosed brother or sister. The siblings have developed several coping strategies *(theme 2)* to deal with the behavior of the diagnosed child. Sometimes these strategies seem unconscious and instinctive. Accepting the fact that their brother or sister is different seems difficult for the siblings, which is a recurring theme. Coping strategies can include seeking support from the parents or ignoring the behavior of the diagnosed brother or sister. Other reactions can be more impulsive; for example, some siblings get aggressive themselves.

Despite the appreciation of joint family activities, the siblings also value having time for themselves *(theme 3)*. They describe that they want to have the opportunity to spend time alone with their parents and express a desire to have a place of their own in the home space. They mention that when the brother or sister is not at home, they experience receiving greater attention from their parents. Parents often—mostly unwittingly—seem to expect the siblings to meet the needs of their brother or sister, which often means that siblings need to sacrifice their own needs, space and time.

Talking about problems, conflicts and negative emotions with trusted people in their personal circle *(theme 4)* is seen as valuable and supportive. In the first place, the siblings go to their parents for support and help. Other significant and trusted persons can also provide support to the siblings, such as other family members, friends or teachers. Although the siblings understand their brother or sister needs more attention or care and they often see the necessity of this, it can be frustrating to feel that they are not being treated equally. In general, there does not seem to be a need for professional help. Some siblings had experience talking to a psychologist or parent mentor. Professional help can be counterproductive when siblings experience too much attention and feel that their problem gets too loaded. Some (younger) siblings mention they would rather play than talk about these themes, indicating that a playful support could be more effective.

The narratives also reveal that the siblings wish their lives could be as “normal” as possible, and that they feel the need to be just like other children *(theme 5)*. They often try to behave as normal as possible with their brother or sister, trying to avoid negative interactions. Some siblings fantasize about what their lives would be like without having a brother or sister with a psychiatric disorder.

Some of the siblings mention that their brother or sister with a psychiatric disorder influences the course of their personal life and the family life *(theme 6)*. One sibling, for example, describes that having sisters with autism made her a different person and influences her future choices. For instance, she explains that she would be less organized without her sisters. Another sibling mentions that a lot of choices within the family (i.e., the type of activities they choose to do as a family) are made based on the needs of her diagnosed sister.

As previously mentioned, participating in joint family activities and sharing positive experiences are valued a lot *(theme 7)* by the siblings. However, the siblings realize that some types of play cannot involve their diagnosed brother or sister. There often is a discrepancy between what the sibling would like to do and what the diagnosed brother or sister likes to do. Most of the time the needs or restrictions of the brother or sister determines what the activity will be and the sibling again has to adjust.

In each interview the respondents were asked what they found helpful in dealing with their brother or sister *(theme 8)*. Despite mentioning they do not need professional help, some siblings received useful advice from professionals. Although the way in which the siblings interact with their diagnosed brother or sister differs a lot between individuals, some forms of communication were recurring. Clarity is important for effective and pleasant communication. It also helps to have a calm and positive attitude toward the diagnosed brother or sister. Ignoring the disruptive behavior can be useful, as well as not taking verbal aggression personally. Other advice given were handling conflicts as a game or using a reward system.

The last main theme explores the ambivalent feelings the siblings experience (in relation to their brother or sister) *(theme 9)*. The siblings love their brother or sister, but at the same time find the fights and the disruptive behavior difficult. They like it when their brother or sister is away from home, because that is when they receive more attention. At the same time, they miss their brother or sister when they are away from home. Since the siblings feel very loyal to their brother or sister as well as their parents, they can feel guilty when they experience negative feelings about their brother or sister. Some of the siblings mentioned that they do not mind that most of the attention of their parents is going to their brother or sister, which can also indicate a fear of disloyalty.

### Latent Dirichlet Allocation

The interviews consisted of a total of 50,632 words with an average of 3,894 words per interview. After the preprocessing and filtering, the corpus consisted of 7,831 terms appearing in 1,492 documents (separate answers). The frequently occurring words that were manually filtered because they did not attribute to forming topics are listed in Supplementary Table 7 in the Supplementary Material. The most frequently occurring unigrams of the corpus are listed in Table 4. It appeared that, within the range of number of topics that the coherence score was calculated for (15 to 25 topics), the model with 24 topics performed the best. The manually inspected models with 10, 30 or 40 topics produced topics that were either incohesive or seemed to fuse more than one topic into a single topic.

**Table 4:** Ten most frequent unigrams from the terms selected for the LDA model.

	**English unigram**	**Dutch unigram**	**Term frequency**
1	Going	Gaan	564
2	Saying	Zeggen	237
3	Finding	Vinden	212
4	Thinking	Denken	161
5	Nice	Leuk	157
6	Knowing	Weten	157
7	Mama	Mama	144
8	Sitting	Zitten	143
9	Good	Goed	141
10	Angry	Boos	119

The three assessors found consensus over the interpretation of the topics in the model, scoring 45,8% of the topics as easily intelligible, 37,5% as difficult to interpret and 16,7% as not intelligible. For example: the label *parents* was assigned to a topic consisting of the uni- and bigrams “mama, papa, going papa_mama, knowing, room, working, saying, going_mama, papa_going, going_papa, mama_papa, going_room, mama_going, mama_saying, going_going, leaving, telephone, conversations”. An overview of the 24 derived topics can be seen in Table 5, including their labels, their probable prevalence in the set of interviews and their quality of intelligibility. Table 6 lists the complete top 20 of probable topic words for the three most prevalent topics. As the interviews were conducted in Dutch, the terms used for the analysis were Dutch as well. For the purpose of this paper, the terms were translated to English. In some cases, it was chosen to translate the words so that the meaning was accurately reflected rather than opting for the literal translation. For example, “rekening_houden” will literally translate to “account_keeping”, while in Dutch the combination of this bigram means “take into account”. The most frequently occurring topics in the interviews were “parents” (5.3%), “(enjoying) sports” (5.3%), “going well” (4.7%), “opinion (I)” (4.6%) and “anger at home” (4.5%).

**Table 5:** List of calculated LDA topics along with their prevalence and intelligibility.

**Topic label**	**Prevalence (%)**	**Intelligibility**
Parents	5.3	+
(Enjoying) sports	5.3	+−
Going well	4.7	+−
Opinion (I)	4.6	+
Anger at home	4.5	+
Fight	4.5	+
Giving attention	4.5	+−
Vacation	4.2	+−
*Not intelligible*	4.2	−
(Bed)room	4.2	+−
Talk to understand	4.2	+−
Siblings	4.2	+
Social network	4.1	+−
*Not intelligible*	4.0	−
Opinion (II)	3.9	+−
Emotion	3.9	+
School	3.9	+
*Not intelligible*	3.9	−
*Not intelligible*	3.8	−
Medication	3.8	+
Nice things	3.7	+
At home and out	3.7	+
Leaving alone	3.6	+−
Taking into account	3.3	+

**Table 6:** Three most frequently occurring LDA topics and the top 20 words in the topic.

	**Topic label**	**Prevalence (%)**	**Intelligibility**	**Top 20 unigrams and bigrams**
15	Parents	5.3	Good	mama, papa, going, papa_mama, knowing, room, working, saying, going_mama, papa_going, going_papa, mama_papa, going_room, mama_going, mama_saying, going_going, leaving, telephone, conversations
5	(Enjoying)	5.3	Medium	finding, nice, finding_nice, nice_finding, knowing holding, sporting, nice_going, kind, knowing_finding, going_nice, finding_finding, swimming, finding_mama, saying_nice, opening, full, saying_papa, mama_nice exciting
	sports		
3	Going well	4.7	Medium	good, going, going_good, sitting, good_going, weekend, sense, staying, changing, time, atmosphere, day, hard, knowing_good, taling, sense_going, leaving, playing, mistake, one

*The words in the topics are translated from Dutch*.

### Comparison of Qualitative Analysis and LDA

Of the 24 LDA topics, 21 were related to the themes formed by the hermeneutic phenomenology analysis. In Table 3, the 21 LDA topics are listed next to the related manually uncovered theme. The three topics that were not specifically mentioned in the main- and subthemes of the hand-coded approach were “opinion (I)”, “medication” and “school”. Reversely, most themes and subthemes could be linked to one or more LDA topics. However, the LDA topics mostly failed to completely cover the spectrum of the subthemes in the main themes. Moreover, no LDA topic seemed to relate to the main theme “wish for normality”.

## Discussion

### Key Findings

The 13 participants between 8 and 15 years old were able to describe their experiences quite well and showed a high level of reflection on their experiences of living together with their brother or sister with a neurodevelopmental disorder. This study confirms the growing evidence of the value and importance of listening to the siblings themselves, even when they are relatively young (4, 7, 22, 37). The unstructured interviews created space for detailed exploration of the experiences of the siblings in which the children were given the opportunity to decide which topics were being discussed. From the richness of information, nine domains emerged as important in the life of the siblings (Table 3). The narratives teach us that having a brother of sister with a neurodevelopmental disorder has a big influence on the lives of the siblings in different ways. The siblings are often continuously thinking about the best way to approach their brother or sister. They have developed several coping strategies to deal with the difficult behavior they are experiencing. Feelings of ambivalence is another recurrent theme in the narratives. Siblings experience frustration and anger but simultaneously feel guilty about these feelings. This can (partly) be explained by feelings of loyalty they experience toward their diagnosed brother or sister, as well as toward their parents. These feelings often cause the siblings to adapt themselves to their brother or sister in order to maintain a stable situation and avoid stress, while overlooking their own needs.

The findings of the qualitative analysis demonstrate the importance of awareness of the needs and desires of siblings. If there is acknowledgment of the feelings and requests of the siblings, they learn to recognize and accept these feelings. Furthermore, the study shows that dedicated support and attention from close relatives, mostly from the parents, is crucial. The narratives also suggest that to improve care it is important to involve siblings in the treatment of their diagnosed brother and sister, so that they gain a better understanding of the disorder and the related behavior of the brother or sister.

Ultimately the results indicate that additional professional support is not always necessary and could even aggravate problems. When support is nevertheless provided the interviews suggest that help should be provided in an informal and playful manner.

The LDA model highlighted three additive themes of importance. Some of the siblings talked about medication and the role it plays for the diagnosed brother or sister. The siblings mentioned their school and whether their brother or sister went to the same school or a different (special) school. A final LDA topic (“opinion (I)”) highlights that the siblings have been talking about the way in which they or other people think about the diagnosed brother or sister. The themes and topics found manually and through machine-learning in this study overlap partially with former research that has been done to evaluate the quality of life of siblings with an autism spectrum disorder by Moyson and Roeyers (7), such as the need for taking time for oneself, shared family activities and social support.

### LDA Topic Model vs. Qualitative Analysis

The LDA model performed moderately when analyzing these narratives. The model produced topics of which the majority were intelligible. However, only 45.8% of the topics were easily intelligible, while the rest of the topics lacked an undisputed interpretability. The calculated cohesiveness score did not seem to match the level of intelligibility by assessors, so for now we can conclude that the selection of good topics cannot be done fully automatically. The automatically identified topics often were too unspecific. For example, the topic “going well” probably indicates that the siblings talk a lot about things that are going well in their narratives, but the model lacks a way of indicating which things the siblings specifically talk about. Another topic is “attention”, where it is unclear if the attention relates to the sibling or the diagnosed brother or sister (or both). Moreover, it was found that considering bigrams created a lot of comparable terms in a topic (i.e., papa, mama, papa_mama and mama_papa). In summary, the LDA model creates generic themes, but doesn't provide the meaning or context of themes like a qualitative analysis does. The benefit of the LDA model is that it gives the assessor a rudimental and practical insight into the broader topics that are discussed as well as the distribution of the topics over the interviews. It also offers a more neutral approach than a qualitative analysis, which deals with bias formed by the perspective or interpretation of the researcher. Furthermore, the LDA model calculated three topics that were not specifically addressed in the qualitative analysis (i.e. opinion, medication, school), suggesting that the latter can overlook certain themes. Ultimately, the LDA analysis is a lot quicker than the qualitative analysis. On the other hand, it was clear that the LDA model did not provide the details and meaning that one can find with a qualitative analysis. The results are line with the study by Howes et al. (28) who compared hand coded topics and LDA topics in therapy dialogues in adults.

### Limitations

One of the main limitations of the study us that the number of participants in this study is small (*n* = 13). It has been suggested that when dealing with a homogenous population saturation (the point at which no new information or themes in the data will be observed) is presumably attained after 12 interviews (38). Since our population was relatively homogenous, adding more interviews might not have contributed to finding more themes, but it might have provided more detailed information related to the identified themes. Moreover, in qualitative research the attention lies on the individual stories. Every story is unique and generates examples of general phenomena, meaning further research with more narratives could undoubtedly provide further insights. Compared to other LDA studies the dataset was not large, which probably has contributed to the percentage of less intelligible topics being produced by our model (26, 28).

Another limitation of the study was the fact that all participants were recruited in the region of UMC Utrecht (Utrecht, The Netherlands); this could cause bias considering the fact that the mental health system might be organized differently in other regions.

Moreover, the diversity of psychiatric disorders of the brothers and sisters of the siblings in the research was limited e.g., none of the siblings had a brother or sister diagnosed with psychosis or anxiety disorder. Although developmental disorders (e.g., hyperactivity disorder, autism spectrum disorder) or behavioral disorders are the most common psychiatric disorders in (young) children, we are aware that siblings of children with other disorders might have different experiences and needs.

Another limitation of the study is that a general question list was used so that the children would feel comfortable and start talking to the interviewer. This may have contributed to the formation of certain topics (in both the qualitative as well as the machine learning analysis).

### Future Recommendations

We suggest that extensive research of siblings' narratives could attribute to a better understanding of their experiences and needs. Future research is above all needed to investigate the experiences and perspectives from siblings of children with different psychiatric disorders, such as anxiety or psychosis. We can assume that these siblings will have different needs for help or support, since every psychiatric disorder has specific characteristics. This also applies to children from different cultures or different living environments. In this study, children from different age groups have not been examined separately, but this could be interesting for further research. It is likely that siblings' experiences and their needs for help and support will vary depending on their stage of development.

For the use of machine learning in the analyzing narratives, we would recommend using a larger dataset to gauge whether the topics get more intelligible. More LDA analyses of narratives of experiences in mental health could also contribute to the evaluation of the validity and feasibility of the model. In addition, when more narratives of siblings are collected, the LDA model could be used to classify new narratives and make predictions, like Valenti et al. (29) did in their study. This could differentiate between different types of siblings and predict what kind of support they need (or do not need). This study also suggests that the LDA model could be a helpful tool in future qualitative studies of narratives. The first part of the hermeneutic phenomenology consists of a global reading and orientation of the interviews (naïve reading). Adding machine learning at this stage could help in obtaining a reliable overview of the discussed themes to further analyze.

### Summary

In summary, the present study found that studying narratives of siblings of children with a neurodeveloptmental disorder contributes to a better understanding of the experiences and needs of siblings. Siblings cope with ambivalent feelings toward their brother or sister, and this emotional conflict often leads to behavioral and emotional adjustment of the siblings. Several coping strategies are developed to deal with the behavior of their diagnosed brother or sister like seeking support or ignoring their sibling. Devoted support, time and attention from close relatives is needed. Caregivers should be aware of the situation of a sibling and evaluate if there is an indication for additional professional support. Furthermore, the LDA analysis of the narriatives wasn't able to provide context or meaning to the topics it found like the qualitative analysis did. It was proposed that unsupervised machine learning (LDA) could be a valuable addition to the qualitative analysis with hermeneutic phenomenology by finding overlooked topics and giving a global overview of topics and its distribution in narratives.

## Data Availability Statement

The raw data supporting the conclusions of this article can be made available after contact with the authors and subject to the Ethics Committee.

## Ethics Statement

The studies involving human participants were reviewed and approved by Medical Ethical Commission of the UMC Utrecht. Written informed consent to participate in this study was provided by the participants' legal guardian/next of kin.

## Author Contributions

JB: designed the machine learning experiment, computed the machine learning analysis, and co-wrote the paper. EV: designed the study, conducted interviews, did the qualitative analysis, and co-wrote the paper. KD: involved in the developing of the machine learning script. FS: supervised the research. All authors edited the paper and have read and approved the final manuscript.

## Funding

This project was funded by the University Medical Center Utrecht.

## Conflict of Interest

The authors declare that the research was conducted in the absence of any commercial or financial relationships that could be construed as a potential conflict of interest.

## Publisher's Note

All claims expressed in this article are solely those of the authors and do not necessarily represent those of their affiliated organizations, or those of the publisher, the editors and the reviewers. Any product that may be evaluated in this article, or claim that may be made by its manufacturer, is not guaranteed or endorsed by the publisher.
