# Supplementary material for: Experiences of Siblings of Children With Neurodevelopmental Disorders: Comparing Qualitative Analysis and Machine Learning to Study Narratives
Source: Front Psychiatry. 2022 Apr 28;13:719598. doi: 10.3389/fpsyt.2022.719598 (PMC9096451; doi:10.3389/fpsyt.2022.719598)
Supplement: Supplementary file 1 [file Table_1.DOCX]

**Appendix: Topic list**

1. **Original (Dutch)**

**Inleiding (meestal samen met ouder(s) );**

Korte uitleg van het doel van het onderzoek + mijzelf voorstellen+ gang van zaken interview. Zijn er nog vragen/ zaken waar ik rekening mee moet houden ?

Start geluidsopname.

- Leeftijd?
- Groep en klas? School?
- Broers/zussen?
- Wie wonen er bij jullie thuis?
- Wat zijn je hobby’s? Waar wordt je blij van? Wat zijn jou wensen? Wie is jou voorbeeld?

1. **Hoofdvraag;**

Kun je mij iets vertellen over school of hobby’s?

**Eventuele hulpvragen;**

- Hoe gaat het op school?
- Wat vind je leuk om te doen, zoals hobby’s, spelletjes?
- Doe je veel samen met je broer/zus? Wat dan?
- Hoeveel vrienden heb je?
  - Evt; Wordt je wel eens geplaagd?
  - Evt; vind je het leuk om alleen te spelen of liever met anderen?

1. **Hoofdvraag;**

Kun je mij iets vertellen over je gezin/familie?

Kun je mij iets vertellen wat je leuk en misschien lastig vind aan je broer en zus?

**Eventuele hulpvragen;**

- Hoe ga je met je broer of zus om? / Hebben jullie vaak ruzie? Spelen jullie graag samen?
- Wat vind je leuk of minder leuk aan jou gezin? (contact ouders en onderlinge gezinsleden)
- Hoe gaan je ouders met jou en je broer/zus om? Is dit anders? (Aandacht?)
- Alle kinderen hebben wel eens ruzie met ouders/broers/zussen; hoe is dat bij jullie? Wat gebeurt er dan?
- Alle vaders en moeders zeggen wat ze wel of niet goed vinden; hoe streng vind je jou ouders? Ben je het daarmee eens/oneens? Krijg je wel eens straf?
- Wat doe je samen met je broer, zus, vader en moeder? Spelletjes, praten..

1. **Hoofdvraag;**

Kun je mij iets vertellen over hoe jij je voelt? Maak je je bijvoorbeeld wel eens zorgen of heb je wel eens rare gedachten die je niet uit je hoofd krijgt?

**Eventuele hulpvragen;**

- Wanneer je jou gevoel een cijfer van 0-10 zou geven waarbij 10 het hoogste is, wat zou dit dan zijn?
- Voel je je anders dan klasgenoten/ leeftijdsgenoten (vriendjes/vriendinnetjes)?
- Iedereen is wel eens bang voor iets; waar ben jij bang voor? Sommige kinderen voelen zich wel eens zenuwachtige of gespannen; hoe is dat bij jou?
- Kinderen piekeren wel eens of maken zich zorgen (bijvoorbeeld over dat er iemand ziek kan worden). Maak je je wel eens zorgen ergens over?
- Voel je je wel eens rot of verdrietig/eenzaam?
  - Evt; hoe vaak? Bij alles wat je doet? Weet je hoe dit komt?

1. **Hoofdvraag;**

Kun je iets bedenken wat jou hulp geeft in jou leven? Waar wordt je blij van in je leven en wat vind je moeilijk?

**Eventuele hulpvragen;**

- Waar wordt je blij van in jou gezin?
- Naar wie ga je toe als je hulp nodig hebt of niet zo lekker in je vel zit?
- Wat vind je het moeilijkste aan jou broer/zus?
- Wat vind je het moeilijkste in jou gezin?
- Heb je tips voor andere broers of zussen?

**Tot slot;**

- Zijn er nog vragen?
- Wil je nog iets vertellen?

Samen het toestemmingsformulier invullen.

**Checklist**;

- Leef/woon/gezinssituatie
- Opleiding; school, klas, niveau
- Leeftijd broer/zus met psychische aandoening
- Aandoening broer/zus met psychische aandoening.
- Eigen leeftijd

**2) English translation**

**Introduction (mostly together with parents):**

Short explenation of the purpose of the study + introducing myself + what the interview will be like. Are there questions? Things we should reckon with?

Start audio recording.

- Age?
- Class? School?
- Siblings?
- Who live at your home?
- What are your hobbies? Wat makes you happy? What are your wishes? Who is your example?

1. **Main question**

Can you tell me something about school or hobbies?

**Optional help questions:**

- How are things going at school?
- What do you like to do, like hobbies, games?
- Do you do a lot together with your brother/sister? Like what?
- How many friends do you have?
  - Optional; are you being bullied?
  - Optional: do you like playing alone or do you rather play with others?

1. **Main question**

Can you tell me something about your family?

Can you tell me something that you like and perhaps find difficult about your brother and sister?

**Optional help questions;**

- How do you interact with your brother or sister? / Do you fight a often? Do you like playing together?
- Wat do you like or like less about your family? (Contact with parents and between family members)
- How do your parents interact with you and your brother/sister? Does that differ? (Attention)
- All children have a fight sometimes with parents/brothers/sisters; how is that with you? What happens then?
- All fathers and mothers say what they approve and what they don’t; how strict do you rate your parents? Do you agree with that or not? Do you get punishment sometimes?
- What do you do together with your brother, sister, father and mother? Games, talking…

1. **Main question;**

Can you tell me something about how you feel? For example, do you sometimes worry or do you have werd thoughts that you cannot get out of your head?

**Optional help questions:**

- Wanneer je jou gevoel een cijfer van 0-10 zou geven waarbij 10 het hoogste is, wat zou dit dan zijn?
- If you would have to rate how you are feeling from 0 to 10, and 10 is the highest, what would it be?
- Do you feel different from you classmates/peers (friends)?
- Everybody is scared of something sometimes; what are you afraid of? Some children are feeling nervous or stressed sometimes, how is that for you?
- Children worry sometimes (for example that someone can get sick). Are you worrying somethings about something?
- Do you ever feel bad or sad/lonely?
  - Optional: how often? With everything you do? Do you know what causes this?

1. **Main question;**

Can you think of something that helps you in your live? What makes you happy in your life and what do you find difficult?

**Optional help questions:**

- What makes you happy in your family
- Who do you go to if you need help or if you’re not feeling well?
- Wat do you find the most difficult about your brother/sister?
- What do you find the hardest about your family?
- Do you have recommendations for other siblings?

**Finally;**

- Are there other questions?
- Is there something you still want to tell me?

Fill in the consent form together.

**Check list**;

- Situation of living/family situation
- Education, class, level
- Age brother/sister with psychiatric disorder
- Type of psychiatric disorder of sibling
- Age
